# LC-MS based urine untargeted metabolomic analyses to identify and subdivide urothelial cancer

**DOI:** 10.3389/fonc.2023.1160965

**Published:** 2023-05-12

**Authors:** Ming Yang, Xiaoyan Liu, Xiaoyue Tang, Wei Sun, Zhigang Ji

**Affiliations:** ^1^ Department of Urology, Peking Union Medical College Hospital, Chinese Academy of Medical Science, Peking Union Medical College, Beijing, China; ^2^ Core Facility of Instrument, Institute of Basic Medical Sciences, Chinese Academy of Medical Sciences/School of Basic Medicine, Peking Union Medical College, Beijing, China

**Keywords:** urine metabolomics, bladder cancer, LC-HRMS, biomarkers, upper tract urothelial cancer, liquid biopsy

## Abstract

**Introduction:**

Urine metabolomics has been a promising technique in the liquid biopsy of urothelial cancer (UC). The comparison of upper tract urothelial cancer (UTUC), lower tract urothelial cancer (BCa), and healthy controls (HCs) need to be performed to find related biomarkers.

**Methods:**

In our investigation, urine samples from 35 UTUCs, 44 BCas, and 53 gender- and age-matched HCs were analyzed using liquid chromatography-high resolution mass spectrometry (LC-HRMS). In different groups, the differential metabolites and the disturbed metabolism pathways were explored. Transcriptomics and urine metabolomics are combined to identify the probably disturbed gene in BCa.

**Results:**

With an area under the curve (AUC) of 0.815, the panel consisting of prostaglandin I2, 5-methyldeoxycytidine, 2,6-dimethylheptanoyl carnitine, and deoxyinosine was able to discriminate UC from HCs. With an AUC of 0.845, the validation group also demonstrated strong predictive ability. UTUC and BCa without hematuria could be distinguished using the panel of 5'-methylthioadenosine, L-beta-aspartyl-L-serine, dehydroepiandrosterone sulfate, and N'-formylkynurenine (AUC=0.858). The metabolite panel comprising aspartyl-methionine, 7-methylinosine, and alpha-CEHC glucuronide could discriminate UTUC from BCa with hematuria with an AUC of 0.83. Fatty acid biosynthesis, purine metabolism, tryptophan metabolism, pentose and glucuronate interconversions, and arachidonic acid metabolism were dysregulated when comparing UC with HCs. PTGIS and BCHE, the genes related to the metabolism of prostaglandin I2 and myristic acid respectively, were significantly associated with the survival of BCa.

**Discussion:**

Not only could LC-HRMS urine metabolomic investigations distinguish UC from HCs, but they could also identify UTUC from BCa. Additionally, urine metabolomics combined with transcriptomics can find out the potential aberrant genes in the metabolism.

## Introduction

Urothelial cancer (UC) is one of the most prevalent malignancies of the urinary system, which mainly includes lower tract urothelial cancer (bladder cancer, BCa) and upper tract urothelial cancer (UTUC) (1). Bladder cancers are the most common type of UCs. UTUCs account for 20-30% of UCs in China with the rate in Western countries lower at 5-10% (2). In 2012, there were an estimated 430 000 new bladder cancers diagnosed worldwide (3). Besides, the annual incidence of UTUC is nearly two cases per 100,000 people. The gold standard to diagnose UTUC and BCa is using a diagnostic endoscopy in combination with a histological biopsy. However, these are intrusive exams that might result in urinary tract infections and physical discomfort for patients. Additionally, bladder recurrence in UTUC has been linked to ureteroscopy (4). The prognostic imaging tests most frequently utilized are CT and ultrasonography. But they still have limitations in detecting tumors with small sizes (5). Urine cytology can identify aberrant urinary cells to diagnose UC, but it cannot confirm whether cells originated from UTUC or BCa.

Non-invasive, convenient, and economical diagnostic techniques are required in clinical work to identify UC (UTUC and BCa) and distinguish UTUC from BCa with ease. A series of urine-based techniques have been explored. Although the FDA has authorized some protein- and cell-based diagnostic techniques for the diagnosis of UC including NPM22, BTA, ImmunoCyt, and FISH, these techniques are unable to distinguish between UTUC and BCa (6).

Metabolomics has been widely applied to reveal altered metabolites and linked metabolic pathways in tumorigenesis (7), mainly because it magnifies the minimal changes from the interaction of genome and environment in diverse pathologic states of disease. Considering that urine directly contacts the urothelial cancer cells, the metabolites released by the cancer cell might be enriched in the urine. Besides, the collection of urine is easy and non-invasive. These all lead to urine metabolomics being a relatively promising method to diagnose and subdivide urothelial cancer disease. Regarding bladder cancer, early in 2010, Pasikanti et al. analyzed 24 BC patients and 51 non-BC controls using gas chromatography and time-of-flight mass spectrometry, and they found that 15 metabolites might discriminate between BC and non-BC patients (8). So far, numerous researcher has explored the urine metabolomics of BCa (9). In 2018, Loras et al. assessed the metabolomics of urine in 31 BCa patients both before and after surgery using high-performance liquid chromatography-mass spectrometry (UPLC-MS), they found that the metabolism of phenylalanine, arginine, proline, and tryptophan was perturbed (10). In 2022, wang et al. analyzed urine from 29 BCa and 15 noncancerous controls using UPLC-MS and generate a panel consisting of 11 metabolites that have an AUC of 0.983 to distinguish BCa from controls (11).

However, only a few metabolomics research focused on the diagnosis of UTUC and discerning different metabolites between UTUC and BCa. In 2015, Li et al. applied ^1^H nuclear magnetic resonance (^1^H NMR) to profile metabolites of 39 UTUC patients and 34 healthy controls. Different levels of lactate, creatinine, glutamine, taurine, etc. are found in the serum when compared between the two groups (12). In 2022, Tsui et al. analyzed 22 UTUC, 41 BCa, and 13 control patients’ urine samples using NMR spectroscopy and found that 7 metabolites including histidine, valine propylene glycol, glycine, leucine, acetylsalicylate, and isoleucine could discriminate BCa and UTUC separately (13). There remains much need to conduct extensive research utilizing various technique routes and statistical approaches.

Our study focuses on identifying the differential urinary metabolites between UCs(UTUCs and BCa) and healthy controls as well as between UTUC and BCa by LC-HRMS. Each comparison group is gender- and age-matched to reduce disturbance. A total of 132 urine specimens were examined, consisting of healthy controls (HCs, n=53), bladder cancers (BCa, n=44), and upper tract urothelial carcinomas (UTUCs, n=35). Furthermore, considering the diagnostic utility of urine metabolomics may also be influenced by hematuria (7, 14), we compare UTUC and BCa with hematuria and without hematuria respectively. To improve diagnosis efficacy, a combinatorial biomarker panel was designed for each group. Additionally, urine metabolomics and transcriptomics are integrated to investigate if specific genes originating from different metabolites have prognostic significance for BCa patients.

## Methods

### Patients and sample collection

This study was approved by the Institutional Review Board of the Institute of Basic Medical Sciences and Peking Union Medical College Hospital, Chinese Academy of Medical Sciences. Before taking part in this study, all the subjects signed written informed consent. Urine samples were taken from Peking Union Medical College Hospital for both cancer patients and healthy controls. The postoperative pathology determined the diagnoses for all of the patients. The following conditions were excluded: severe hepatic and renal dysfunction, malnutrition, and metabolic diseases like diabetes. After an overnight fast, midstream urine was collected in the morning between 6:30 and 9:00 to eliminate dietary disturbance. Within six hours, all samples were centrifuged followed by the separation, aliquotation, and storage of the supernatants at -80°C until analysis. Haematuria is characterized by the presence of more than 3 red blood cells per high-power field in centrifuged urine sediment examined under a microscope.

### Urine sample preparation

A previously utilized procedure was applied to prepare the urine sample (15). In short, Each 200 uL urine sample was mixed with 200 uL of acetonitrile, and the mixture was vortexed for 30 seconds before being centrifuged at 14,000 x g for 10 minutes. After being vacuum-dried, the dried powder was dissolved with 200 uL 2% acetonitrile. Using 10 kDa molecular weight cutoff ultracentrifugation filters, urine metabolites were separated from larger molecules before being delivered to autosamplers (Millipore Amicon Ultra, MA). The quality control (QC) specimens were pooled urine specimens which were created by combining aliquots of all specimens from various analytical groups. As a result, it can comprehensively represent the entire sample set. During the analytical run, the QC samples were injected every 10 samples to provide a set of data and confirm the method’s repeatability and stability.

### LC-HRMS analysis

A Waters ACQUITY H-class LC system coupled with an LTQ-Orbitrap mass spectrometer (Thermo Fisher Scientific, MA, USA) was utilized to analyze the urine samples. To separate urine metabolites, a 17-minute gradient was applied to a Waters HSS C18 column (3.0x100 mm, 1.7 um) at a flow rate of 0.5 ml/min. Mobile phase B consisted of acetonitrile, whereas mobile phase A involved 0.1% formic acid in water. Followed were the gradient’s setting:0-2 min,2% solvent B;2-5 min, 2-55% solvent B;5-15 min,55-100% solvent B;15-20 min,100% solvent B;20-20.1 min,100-2% solvent B; and 20.1-29 min,2% solvent B. The column’s temperature was kept at 50°C throughout the procedure. The mass scan was in the 100–1,000 m/z range. The MS1 and MS2 analyses were run at resolutions of 60K and 15K, respectively. The MS1 automatic gain objective was set to 1 x 106, and the maximum injection time (IT) was 100 milliseconds. The maximum IT was set to 50ms, and the MS2 automatic gain control target was 5x105. The ideal collision energy of 20, 40, 60, or 80 was employed to dissociate the various metabolites using the higher-energy collisional dissociation (HCD) fragmentation mode. As a result, the features in MetaAnalyst 4.0 (http://www.meta-boanalyst.ca) may be more comparable.

### Data processing of metabolomics

Using the Progenesis QI (Waters, Milford, MA, USA) tool and based on the established identification method (16, 17), we evaluate the mass spectrometry original data. Data management and metabolite identification procedures can be found in **Table S2**. We developed a variety of statistical methods, including Pareto scaling, log transformation, and missing value estimation. Variables lacking in 50% of the samples were not included. Non-parametric tests were employed to evaluate the variables’ significance. Significant data had an adjusted P-value (FDR) less than 0.05. To conduct evaluations of pattern recognition (principal component analysis, PCA; for orthogonal partial least squares discriminant analysis, OPLS-DA), we employed SIMCA 14.0 software (Umetrics, Sweden). Any alteration that meets all the criteria was considered valuable, including Fold change >1.5, adjusted P<0.05, and VIP value >1. To assess the prediction accuracy and using the MetaAnalyst 5.0 platform, we carried out an independent verification and a ROC analysis. An exploratory investigation based on a multivariate ROC curve was used.

### Integration of metabolomics and transcriptomics and survival analysis

RaMP (https://rampdb.nih.gov/) was employed to match changed metabolites between BCa and HCs to their associated genes based on The Human Metabolic Data Base (HMDB). We used transcriptomic profiles of BLCA from The Cancer Genome Atlas (TCGA) to find differential genes between 431 BCa patient tissues and 19 tissues adjacent to cancer by “DESeq2” R package(|log2(FC)|>1, p.adj<0.05). By intersecting the above gene sets, common genes from metabolomics and transcriptomics were identified. The survival status of the common genes was analyzed in TCGA-BLCA by Kaplan Meier curves. “ggplot2” R package and GraphPad Prism (version 9, USA) were used to generate the graphs.

## Results

### Baseline information

35 UTUC patients (19 without hematuria and 16 with hematuria), 44 BC patients (30 without hematuria and 14 with hematuria), and 53 age- and sex-matched healthy controls (HCs) made up the total population of participants in our study. **Table 1** displays the clinical data from all enrolled individuals (Details in **Table S1**).

**Table 1:** The baseline information of all enrolled subjects in the study.

	UC vs. control				BCa vs. UTUTC			
	Discovery group		Validation group		Without hematuria		With hematuria	
	UC	Control	UC	Control	BCa	UTUC	BCa	UTUC
Cases(n)	31	34	18	19	30	19	14	16
Age(yrs)	66.3 ± 10.6	61.7 ± 12.3	66.3 ± 10.7	61.2 ± 11.4	66.1 ± 10.6	64.6 ± 13.1	68.9 ± 10.6	71 ± 9.7
Gender(M/F)	17/14	22/12	9/9	12/7	17/13	10/9	5/9	4/12
Grade(LG/HG)	9/22	NA	4/14	NA	6/24	8/11	1/13	4/12
Stage(Ta-T1,T2-T4)	12/19	NA	10/8	NA	14/16	9/10	3/11	10/6

n, sample size; yrs, years; M, male; F, female; LG, low grade; HG, high grade; UC, urothelial cancer; BCa, bladder cancer; UTUC, upper tract urothelial cancer; NA, Not Applicable; Ta-T1=non-muscular invasive; T2-T4, muscular invasive.

**Figure 1:**
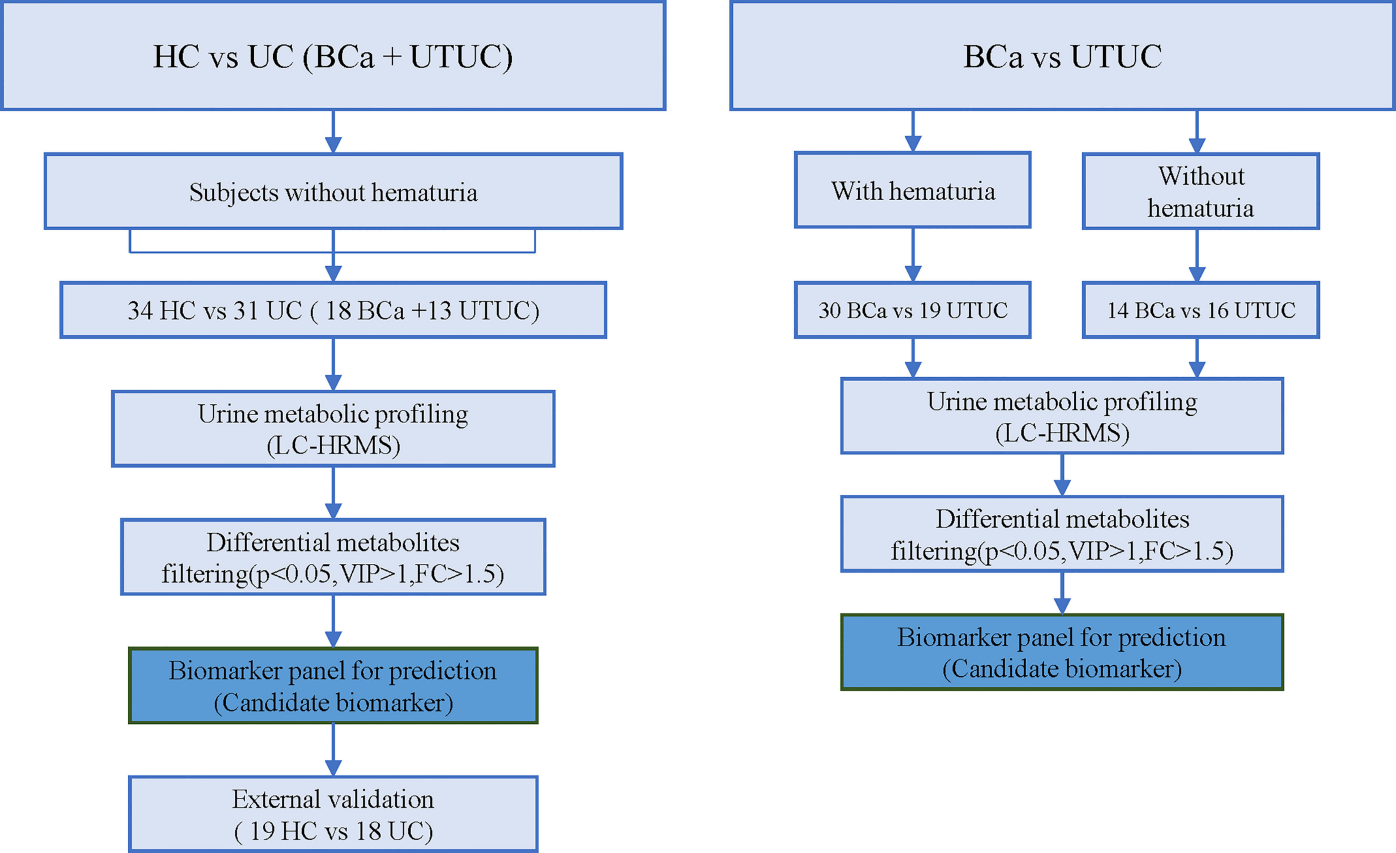
Workflow.

**Figure 2:**
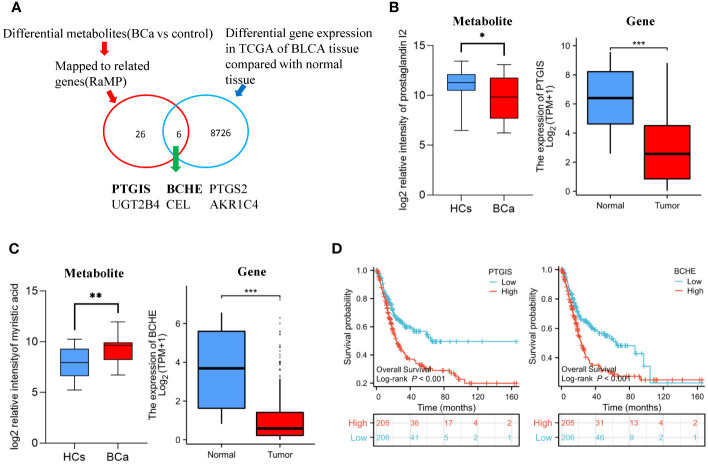
Differential gene discovered by metabolomics in conjunction with transcriptomics **(A)** Overview of the strategy used to profile the differential genes of BCa. **(B)** The relative intensity of prostaglandin I2 in urine metabolomics and the expression of PTGIS gene in TCGA-BLCA cohort **(C)** Relative intensity of myristic acid in urine metabolomics and the expression of BCHE gene in TCGA-BLCA cohort. **(D)** T Survival analysis of PTGIS and BCHE gene in TCGA-BLCA cohort.

Biomarkers for UCs were discovered through the examination of 31 UC and 34 HC specimens. Then, the biomarkers were confirmed in a subsequent batch of validation group that included 18 UC specimens and 19 HC specimens. Subsequently, to identify biomarkers that might discriminate between BC and UTUC, 30 BC specimens and 19 age- and gender-matched UTUC specimens underwent differential examination, and 14 BCa specimens with hematuria were compared with 16 UTUC specimens with hematuria. Furthermore, the metabolic gene associated with differential metabolites between UCs and HCs was determined using HMDB. Integration of metabolite alterations with transcriptome data from TCGA was utilized to identify genes in common. **Figures 1** and **2A** depict the study’s procedure.

The system’s stability was assessed., which is a crucial step in large-scale metabolomic research, using a quality control (QC) sample. The system has strong stability, as shown by the QC correlation (**Figure S1**), which is in the range of 0.97 to 1.

### Analysis of UC vs. the control group

**Figure 3:**
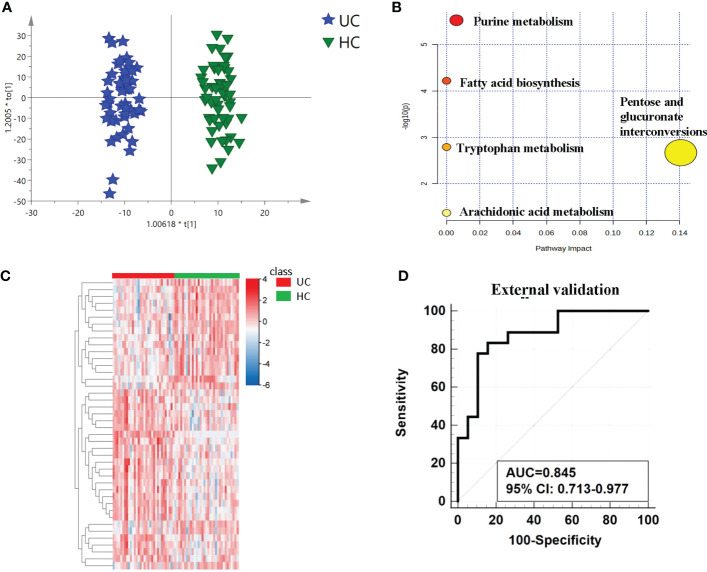
Analysis of metabolic profiling between UCs and controls. **(A)** Metabolic score plot of OPLS-DA. **(B)** Pathway analysis of the differential metabolites between the two groups. **(C)** The relative intensity between the cancers and controls. showed by heatmap. **(D)** ROC curve in the external validation group based on the same biomarker panel.

We used a variety of statistical approaches to evaluate the urine metabolome from UC and HC specimens. The metabolic profiles of UC patients and HC patients demonstrated a discriminatory trend according to PCA (**Figure S2A**). Better separation was established by OPLS-DA (**Figure 3A**), and distinct compounds were chosen by the value important plot value (VIP>1). To ensure that the supervised models were stable and robust, 200 permutation tests were performed (**Figure S2C**). Finally, 42 significantly differential compounds were discovered (**Table S2A**) Heatmap shows the obvious distinction of all the differential metabolites between UC and HC patients (**Figure 3C**).

The differential metabolites’ diagnostic efficacy for UC was assessed using ROC curves. The findings revealed that 28 metabolites with AUC values over 0.7 had potential clinical diagnostic utility, and 3 metabolites with AUC values above 0.8 had better diagnostic value (**Table S2A**). The diagnostic ability of each metabolite in the panel shows in **Table 2** and **Figure S5**. When comparing UC with the control, it was discovered that a panel of metabolites made up of prostaglandin I2, 5-methyldeoxycytidine, 2,6-dimethylheptanoyl carnitine, and deoxyinosine had the highest prediction precision. The AUC was 0.815 for the 10-fold cross-validation (**Figure S2B**). After that, the model underwent an external validation which AUC was 0.845 (**Figure 3D**). The sensitivity and specificity were 0.778 and 0.842 respectively (**Table 3**). When UCs were compared to healthy controls, the pathway analysis data revealed that metabolites were engaged in fatty acid biosynthesis, purine metabolism, tryptophan metabolism, pentose and glucuronate interconversions, and arachidonic acid metabolism. (**Figure 3B**). Statistically significance of the metabolites in different pathway analyses shows in **Table 4**.

**Table 2:** Diagnostic ability of each metabolite in the panel.

Metabolites in panel	AUC	Sensitivity	Specificity	T-test	FC
UC vs controls					
Deoxyinosine	0.81	70.6	80.7	1.8E-05	0.44
2,6 Dimethylheptanoyl carnitine	0.73	79.4	58.1	0.002	0.61
Prostaglandin I2	0.70	82.4	54.8	0.012	0.52
5-Methyldeoxycytidine	0.69	82.4	54.8	0.013	2.01
UTUC vs BCa without hematuria					
5’-Methylthioadenosine	0.87	88.9	83.9	0.027	1.94
L-beta-aspartyl-L-serine	0.86	88.9	64.5	5.53E-06	3.03
Dehydroepiandrosterone sulfate	0.79	88.9	64.5	0.002	0.27
N’-Formylkynurenine	0.75	94.4	54.8	0.005	1.56
UTUC vs BCa with hematuria					
Aspartyl-Methionine	0.84	62.5	100	0.024	3.01
7-Methylinosine	0.73	100	50	0.009	0.48
alpha-CEHC glucuronide	0.73	62.5	78.6	0.049	1.85

**Table 3:** Results of the logistic regression model based on different biomarker panels.

Groups	AUC	Sensitivity	Specificity
^1^UC vs control			
Training/Discovery	0.875 (0.848 ~ 0.902)	0.908 (0.876 ~ 0.941)	0.706 (0.653 ~ 0.760)
10-fold Cross-Validation	0.815 (0.712 ~ 0.918)	0.853 (0.853 ~ 0.972)	0.710 (0.550 ~ 0.869)
External validation	0.845 (0.713 ~ 0.977)	0.778 (0.778 ~ 0.970)	0.842 (0.678 ~ 1.000)
^2^BCa vs UTUC without hematuria			
Training/Discovery	0.922 (0.898 ~ 0.946)	0.852 (0.797 ~ 0.907)	0.803 (0.756 ~ 0.850)
10-fold Cross-Validation	0.858 (0.752 ~ 0.965)	0.833 (0.833 ~ 1.000)	0.806 (0.667 ~ 0.946)
^3^BCa vs UTUC with hematuria			
Training/Discovery	0.882 (0.842 ~ 0.921)	0.757 (0.687 ~ 0.827)	0.921 (0.873 ~ 0.968)
10-fold Cross-Validation	0.830 (0.675 ~ 0.986)	0.750 (0.750 ~ 0.962)	0.929 (0.794 ~ 1.000)

^1^ The biomarker panel: prostaglandin I2, 5-methyldeoxycytidine, 2,6 Dimethylheptanoyl carnitine, Deoxyinosine.

^2^The biomarker panel: 5’-Methylthioadenosine, L-beta-aspartyl-L-serine, Dehydroepiandrosterone sulfate, N’-Formylkynurenine.

^3^The biomarker panel: Aspartyl-Methionine, 7-Methylinosine, alpha-CEHC glucuronide.

**Table 4:** Statistically significance of the metabolites in different pathway analyses when comparing UC with HCs.

Metabolites	Pathway Name	Match Status	p	FDR	Impact
Prostaglandin I2	Arachidonic acid metabolism	1/36	0.013	0.0132	0.000
Myristic acid	Fatty acid biosynthesis	1/47	5.4764E-4	0.003	0.000
beta-D-Glucuronoside	Pentose and glucuronate interconversions	1/18	0.011	0.013	0.14062
Deoxyinosine	Purine metabolism	2/65	0.002	0.004	0.00593
Guanosine					
6-Hydroxymelatonin	Tryptophan metabolism	1/41	0.007	0.011	0.000

### Analysis of BCa vs UTUC group

**Figure 4:**
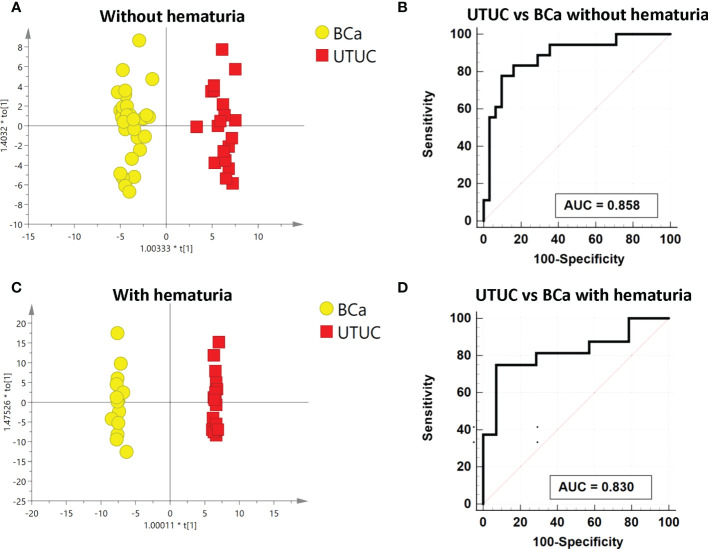
Analysis of metabolic analysis between UTUC and BCa with and without hematuria. **(A)** Metabolic score plot of OPLS-DA between UTUC and BCa without hematuria. **(B)** ROC curve with 10-fold cross-validation based on the biomarker panel between UTUC and BCa without hematuria. **(C)**Metabolic score plot of OPLS-DA between UTUC and BCa with hematuria. **(D)** ROC curve with 10-fold cross-validation based on the biomarker panel between UTUC and BCa with hematuria.

Analysis was also done on the differences between the BCa and UTUC without hematuria groups. The discrimination between the two groups was indicated by PCA and OPLS-DA, as displayed in **Figures S3A** and **4A**, respectively. The model is reliable, as evidenced by 200 permutation tests (**Figure S3B**). As a consequence, 17 substantially different metabolites in total were found (**Table S2B**). The pathway analysis revealed that the metabolism of cysteine and methionine as well as the synthesis of steroid hormones was dysregulated (**Figure S3D**). Thirteen metabolites individually had high diagnostic efficacy with AUC values over 0.7, and three metabolites reported AUC values above 0.8. The heatmap displays the various metabolite concentrations in BCa and UTUC (**Figure S3C**). A reliable panel was established to evaluate if BCa and UTUC without hematuria were comparable using a panel that included 5’-methylthioadenosine, L-beta-aspartyl-L-serine, dehydroepiandrosterone sulfate, and N’-formylkynurenine. In the testing dataset, the panel’s AUC was 0.922, compared to 0.858 in the 10-fold cross-validation (**Figure 4B**). The panel’s sensitivity and specificity were 0.833 and 0.806 respectively (**Table 3**).

To find the differences in metabolic profiles, a PCA model was employed to find the biomarkers which could distinguish between BCa and UTUC with hematuria. **Figure S4A** displays the variation between the two groups. Additionally, a supervised OPLS-DA model was built to further illustrate the distinction (**Figure 4C**). The validated perturbation shows in **Figure S4B**. Based on the VIP of OPLS-DA, it was identified that 17 metabolic molecules in total were significantly different between the two groups (**Table S2C**). The varied metabolite concentrations in these two groups are shown on the heatmap (**Figure S4C**). Four metabolites with an AUC over 0.8 and the other 13 metabolites with an AUC above 0.7, as indicated by the ROC curve, performed separately in distinguishing between BC and UTUC with hematuria. A panel composed of aspartyl-methionine, 7-methylinosine, and alpha-CEHC glucuronide, according to the multivariate ROC curve **Figure 4D**, displayed the strongest ability to discriminate the two groups. AUCs for the 10-fold cross-validation are 0.83 and 0.882 for the discovery group respectively (**Figure S3D**; **Table 3**). Additionally, the sensitivity and specificity were respectively 0.750 and 0.92 (**Table 3**).

### Integration of metabolomics and transcriptomics to identify the BCa gene

There are six metabolites that not only exhibit significant differences between UC and HCs, but also demonstrate notable disparities between BCa and HCs (**Figure S6**). To identify the shared gene, we combined our metabolomics data with publically available transcriptomics data (**Figure 2A**). By using HMDB to map the altered metabolites between BCa patients and healthy controls to their corresponding genes, 26 related genes were found (**Table S3A**). We discovered 8726 genes were significantly different between normal tissues and bladder cancer tissues using the TCGA dataset (**Table S3B**). Six common genes, including BCHE, PTGIS, PTGS2, UGT2B4, and AKR1C4, were observed when the metabolomics-mapped gene set and the TCGA-based gene set were integrated (**Table S3C**). Prostaglandin I2 relative intensity and the expression of its related gene, PTGIS gene, are both decreased in tumor samples (**Figure 2B**). The relative intensity of myristic acid is higher in BCa samples and the expression of its associated gene, BCHE, is lower in the tumor samples (**Figure 2C**). Using the TCGA, we then carried out individual gene survival analysis to gain more understanding of these 6 genes. By using Kaplan-Meier survival analysis, we found that the top 50% expression of BCHE and PTGIS in BCa were both substantially related to poor survival (**Figure 2D**).

## Discussion

Metabolomics is emerging as a potential method in the study of various malignancies in addition to genomics, transcriptomics, and proteomics. The goal of metabolomics research is to develop diagnostic biomarkers for cancer patients as well as to provide new insights into the underlying mechanisms of cancers. Although UTUC and BCa are both urothelial tumors, they have different molecular genotypes. Therefore, the metabolites of the two diseases are expected to differ since metabolomics reflects the downstream results of gene alteration and enzyme function. In this study, we used LC-MS to distinguish between urothelial carcinomas of the upper and lower urinary tract in samples of matched gender and age, with or without hematuria. Besides, combined with urine metabolomics and transcriptomics, the differential genes were investigated when comparing UCs with HCs. Differential candidate metabolites were selected to build the panel in distinct groups based on specific biological functions and disease-related literature, and the disrupted pathway was analyzed.

### UCs vs. healthy controls

In our study, the arachidonic acid metabolism was dysregulated. In 2012, Miryaghoubzadeh et al. applied gas-liquid chromatography between 31 patient-derived bladder cancer tissues and adjacent normal-like tissues. They discovered a significant drop in the level of arachidonic acid in bladder cancer tissues which unveiled the potential effect of the mechanism involved in tumorigenesis (18). In our research, prostaglandin I2 (PGI2), which is catalyzed by prostaglandin I2 synthase (PTGIS) from prostaglandin H2 in the arachidonic acid metabolism (19), was found to have a lower concentration in the urine of UCs than that of healthy controls. We also discovered the reduced expression of the PTGIS gene in TCGA, which to some extent helped explain the lowering concentration of PGI2. PGI2 is considered to have chemopreventive effects in tumorigenesis. According to previous studies, the murine orthodox lung cancer model’s occurrence and progression are inhibited by elevated PGI2 concentration brought on by PTGIS overexpression. After regular exposure to tobacco smoke, 60% of the transgenic murine of the PTGIS gene (9 out of 15) did not develop lung tumors, while the rate in wild-type littermates was 16% (20, 21). In another study, the PTGIS gene is related to bladder cancer’s ability to proliferate, migrate and undergo epithelial-mesenchymal transition (22). Combined with TCGA, we also found out that the PTGIS could predict the prognosis of bladder cancer which further illustrated that the reduction of PGI2 might be one of the results in the UC’s occurrence and development.

Numerous metabolomics investigations in urine, blood, and cell lines of bladder cancer have identified the disrupted purine metabolism (23), which was also observed in our investigation. Deoxyinosine, which participates in the metabolism, has a reduced concentration in the urine of UCs when compared with HCs. Wang et al. reported that deoxyinosine could be the biomarker to differentiate bladder cancer and kidney cancer patients from healthy people (24). Additionally, the deoxyinosine analog, C5-hydroxymethyl-2′-deoxycytidine (5hmdC), is considerably decreased in the renal pelvis of UC patients (25).

It has been found that the examination of DNA adducts and altered DNA bases can help diagnose early cancer (26). The 5-methyldeoxycytidine(5-mdc), also one of the panel, is generated during the process of methylation of cytosine-guanine dinucleotide sequences (CpG dinucleotides) catalyzed by DNA methyltransferase. The detection and quantification is one of the ways to reflect the diverse level of DNA methylation (27). Nishiyama et al. studied genome-wide DNA methylation profiles by comparing 18 normal urothelia from healthy patients with 17 noncancerous and 40 urothelia from UCs, and they found that methylation levels were highest in malignant tissues and lowest in normal tissues. Besides, 9 methylation clones can differentiate whether patients have intravesical metachronous recurrence of UTUC after nephroureterectomy (28, 29). These all indicate that methylation participates in tumor formation and 5-mdc might be an indicator for carcinogenetic risk estimation. It is worth mentioning that as far as we know, it’s the first time 5-mdc was found in the urine of UC patients.

Numerous exogenous toxins, such as aristolochic acid, trigger urothelial cancer. Glucuronidation is crucial for the elimination of toxins, medicines, and other substances that cannot be used as an energy source. Numerous glucuronides are found in our study such as 6-hydroxy-5-methoxyindole glucuronide when UC patients are compared to healthy controls. The rise in the quantity of 6-hydroxy-5-methoxyindole glucuronide can be caused by the increased gene expression of UGT2B4 whose expressed enzyme participates in the metabolism of the glucuronide. According to Dalangood et al., the combination of UGT2B4 and other 13 additional gene signatures may separate the 418 UC patients into 2 groups, which exhibit a statistically significant difference in overall survival (30). Furthermore, an exogenous metabolite called myristic acid, which can be catalyzed by the enzymes bile salt-activated lipase and butyrylcholinesterase (BCHE) encoded by the genes CEL and BCHE respectively is increased in the urine of UC patients. In the TCGA, these 2 genes have varied expression between BC and para-carcinoma tissues. In addition, patients with 50% top and 50% bottom BCHE gene expression differ in terms of overall survival. Cheng et al. also found that myristic acid augmented in the urine low-grade NMIBC samples when compared with the healthy controls (31). Besides, according to Koie et al., the level of serum BCHE following radical cystectomy is a standalone prognostic factor in non-muscle invasive BC patients (32). These all to some extent indirectly suggest the effect of exogenous materials in the development of UC.

### UTUC vs BCa without hematuria

Dehydroepiandrosterone sulfate (DHEAS), one of the metabolites in the panel to distinguish UTUC and BCa without hematuria, is the sulfated form of dehydroepiandrosterone (DHEA). Additionally, DHEAS and DHEA are the most prevalent circulating steroids in humans which engage in the biosynthesis of steroid hormones. The steroid hormone biosynthesis pathway is perturbed in our study, consistent with the result of urine metabolomics research of BCa by Liu et al. and Cheng et al. (15, 31). DHEA given orally to mice can prevent spontaneously induced breast cancer and chemically generated lung and colon cancers (33). Besides, the DHEAS and DHEA are the precursors of androgen and estrogen, and the prevalence of UTUC and BCa in women and men are completely different which can be explained by different levels of gonadal hormones. Male mouse bladder cancer incidence decreases after castration. On the other hand, oophorectomy raises the incidence of bladder cancer in female rats (34). In terms of UTUC, Kawashiwagi et al. immunohistochemically stained 99 UTUC tissues along with matched normal tissues and discovered that the UTUC tissues exhibited considerably reduced levels of androgen receptor (AR), estrogen receptor-α (ERα) and ERβ (35). In summary, the biosynthesis of the steroid hormones plays a significant role in the sex differences in BCa and UTUC. Further research into the role of DHEAS is warranted.

When comparing UTUC with BCa without hematuria, 5’-Methylthioadenosine (MTA), another metabolite in the panel, has an AUC of 0.87 independently. It participates in the metabolism of cysteine and methionine which is dysregulated in our study and the MTA is mainly metabolized by MTA-phosphorylase (MTAP). According to a wide range of studies, MTA may have an impact on cellular gene expression, proliferation, differentiation, and apoptosis (36). Kirovski et al. demonstrated that MTAP gene deletion caused MTA to accumulate, which was related to heightened tumorigenicity (37). Comprehensive genomic profiling of 2,683 advanced and metastatic BC tissue samples was carried out in 2022 by Basin et al. They discovered that, in contrast to MTAP gene intact BC, 650 advanced BC samples (24.2%) had MTAP loss, with frequent co-deletions of CDKN2A/B, co-mutations with FGFR3, and co-mutations with phosphatase and tensin homolog genes, which all contributed to developing new synthetic lethality targeted therapies (38). In our study, the FC(UTUC/BC) of MTA is 1.9. As far as we are aware, no study has focused on the correlation between MTA and UTUC yet, which is worthy of further study.

### UTUC vs BCa with hematuria

Modified nucleotides are a class of nucleotides that are chemically modified in organisms, and these modifications often affect gene expression, protein translation, and other biochemical processes in the organism (39). Instead of being recaptured into mRNA, modified nucleosides can be excreted into the urine. Their metabolic products can be utilized as biomarkers in diagnosing bladder cancer (40). Chang et al. measured 17 nucleosides and they found that m3C and m1A might serve as possible biomarkers for the diagnosis of BCa (41). 7-Methylinosine is detected in our study, which as we know is the first time that the metabolite is found in the urine of UTUC and BCa patients. However, it seems that the nucleosides are universal biomarkers because diverse nucleosides have been used to be biomarkers in different cancer diseases. We, therefore, add alpha-CEHC glucuronide into the panel to improve the AUC when we compare UTUC and BCa with hematuria. Alpha-CEHC combined with glucuronide facilitates its excretion from urine. The alpha-CEHC is the metabolite of vitamin E which not only has the effect against oxidative stress but also can reduce the risk of bladder cancer mortality (42). However, the effect of it on UTUC remains unclear.

In the present study, potential urinary biomarkers and the molecular mechanism behind were tentatively discovered but still need to be thoroughly validated. First, The sample size in our study is relatively small, which may limit the generalisability of our findings. Second, our comparison between UTUC and BCa lacks external validation, which calls for a cautious interpretation of the results. Third, urothelial cancer’s molecular mechanisms are highly complex and intricate. Although we have attempted to elucidate potential molecular mechanisms through the integration of transcriptomics and metabolomics, our explanations remain speculative and lack experimental validation. Future studies should involve larger sample sizes and multicenter collaborations as well as targeted validation of the identified urinary metabolites. Additionally, the molecular mechanisms responsible for the observed perturbations in urinary metabolites should be further investigated and validated through *in vitro* and *in vivo* experiments. This will enable a more in-depth understanding of the metabolic pathways and processes implicated in urothelial cancer development and progression.

## Conclusion

In this investigation, we employ the LC-MS method to identify the panel in the gender- and age-matched UC group, and the impact of hematuria is also taken into account. The findings revealed that the urine metabolome could distinguish between UTUC and BCa with or without hematuria as well as UCs and healthy controls. Combined with transcriptomics we find that the differential PTGIS and BCHE genes, which are related to two metabolites, can predict the prognosis of BCa.

In the future, more centers and patients should be enrolled and the molecular mechanism behind the perturbation of those metabolites is supposed to be validated by *in vitro* and *in vivo* experiments.

## Data availability statement

The datasets presented in this study can be found in online repository (iProX -integrated Proteome resources. ID=IPX0006026000). The names of the repository and URL are as follows: iProX: https://www.iprox.cn/page/project.html?id=IPX0006026000.

## Ethics statement

The studies involving human participants were reviewed and approved by The Institutional Review Board of the Institute of Basic Medical Sciences and Peking Union Medical College Hospital,Chinese Academy of Medical Sciences. The patients/participants provided their written informed consent to participate in this study.

## Author contributions

MY, XL, WS, and ZJ conceived and designed the study. MY and XT collected the clinical data and performed the experiments. MY and XL drafted the first version of the manuscript. WS and ZJ revised the manuscript together. All authors contributed to the interpretation of the results and edited and approved the final manuscript. During the proofing stage, MY compiled all the revisions and uploaded the updated files.
