# Supplementary material for: LC-MS based urine untargeted metabolomic analyses to identify and subdivide urothelial cancer
Source: Front Oncol. 2023 May 12;13:1160965. doi: 10.3389/fonc.2023.1160965 (PMC10226587; doi:10.3389/fonc.2023.1160965)

Supplementary Material

LC-MS based urine untargeted metabolomic analyses to identify and subdivide urothelial cancer

Ming Yang[1], Xiaoyan Liu[2], Xiaoyue Tang[2], Wei Sun[2]* and Zhigang Ji[1]*

[1]Department of Urology, Peking Union Medical College Hospital, Chinese Academy of Medical Science, Peking Union Medical College, Beijing, China

[2]Core Facility of Instrument, Institute of Basic Medical Sciences, Chinese Academy of Medical Sciences/School of Basic Medicine, Peking Union Medical College, Beijing, China

*** Correspondence:** Zhigang Ji[1]*，jizhigang@pumch.cn；Wei Sun[2]*，sunwei@ibms.pumc.edu.cn

Supplementary Figure 1. | Assessment of QC samples.

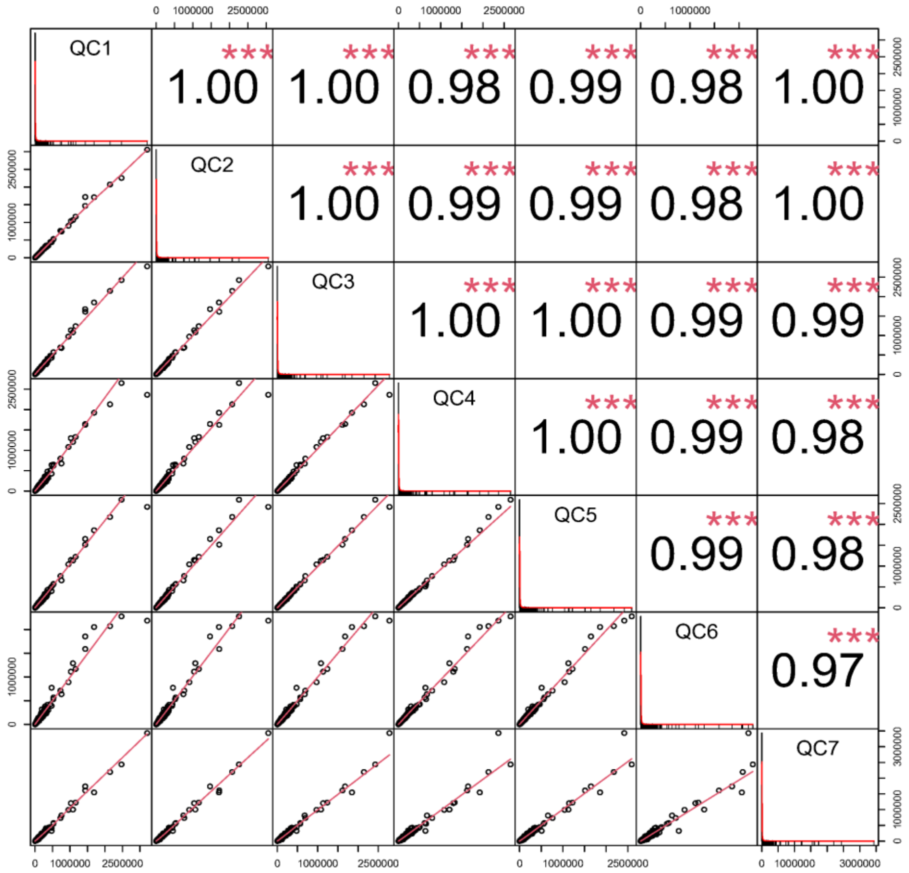

Supplementary Figure 2. | Analysis of metabolic profiling variation of UCs and HCs. **(A).** Score plot of supervised PCA overview of urine metabolic profiling between UCs and HCs. **(B).** ROC curve of the 10-fold cross validation in the discovery group based on the biomarker panel. **(C).** Two hundred permutation tests of the OPLS-DA model based on UCs and HCs urine metabolome.

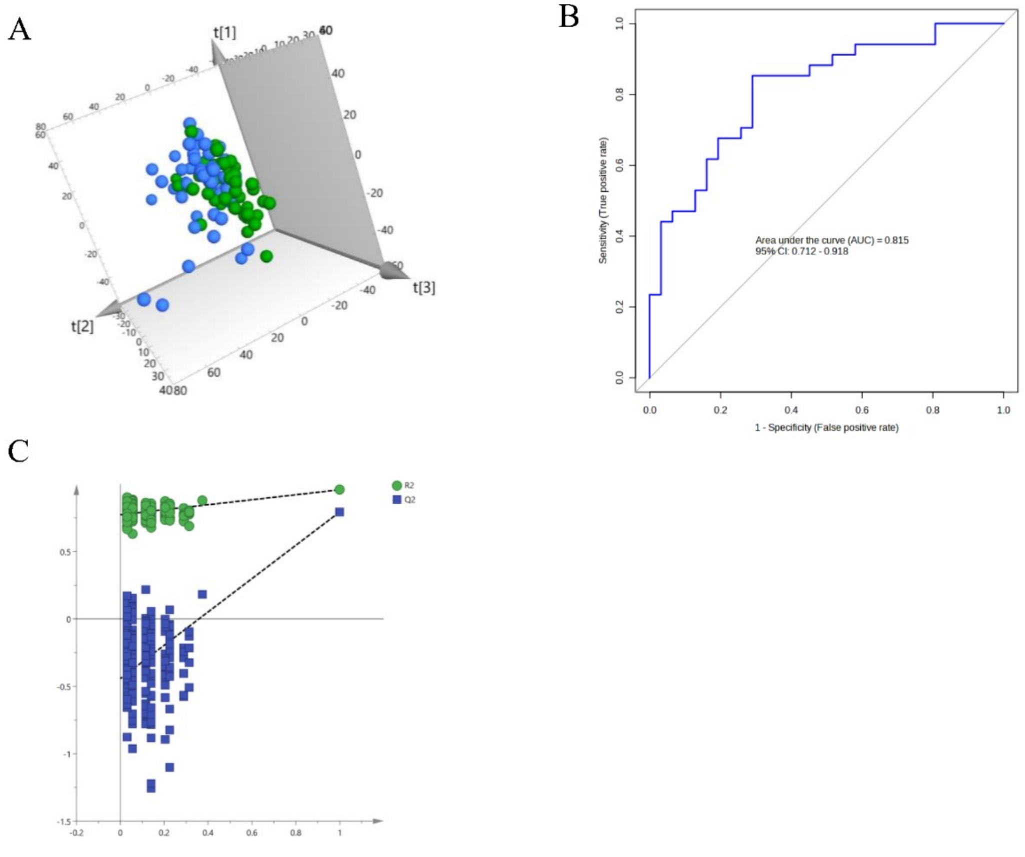

Supplementary Figure 3. | Analysis of metabolic profiling variation of UTUC and BCa without hematuria. **(A).** Score plot of supervised PCA overview of urine metabolic profiling between UTUC and BCa without hematuria. **(B).** Two hundred permutation tests of the OPLS-DA model based on UTUC and BCa urine metabolome. **(C).** The relative intensity of metabolites showed by heatmap. **(D).** Pathway analysis of the differential metabolites between the two subgroups.

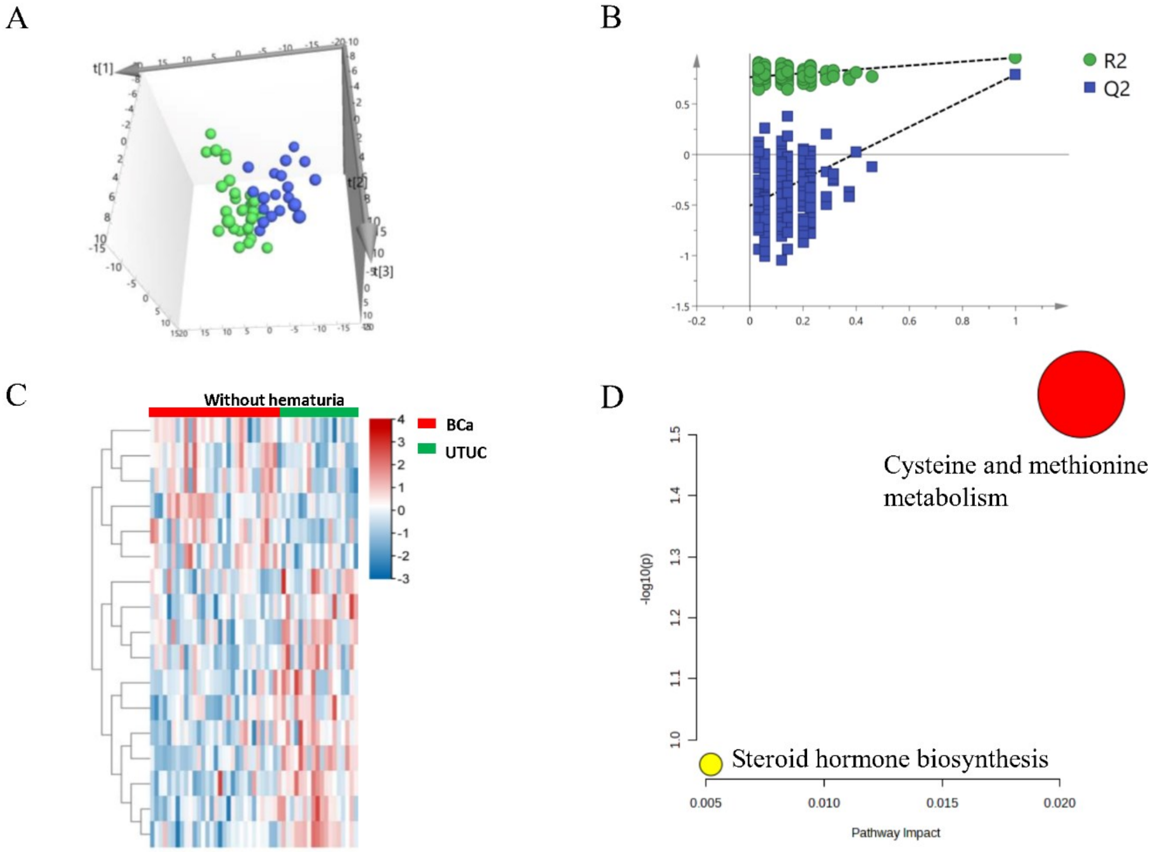

Supplementary Figure 4. | Analysis of metabolic profiling variation of UTUC and BCa with hematuria. **(A).** Score plot of supervised PCA overview of urine metabolic profiling between UTUC and BCa with hematuria. **(B).** Two hundred permutation tests of the OPLS-DA model based on UTUC and BCa urine metabolome. **(C).** The relative intensity of metabolites showed by heatmap.

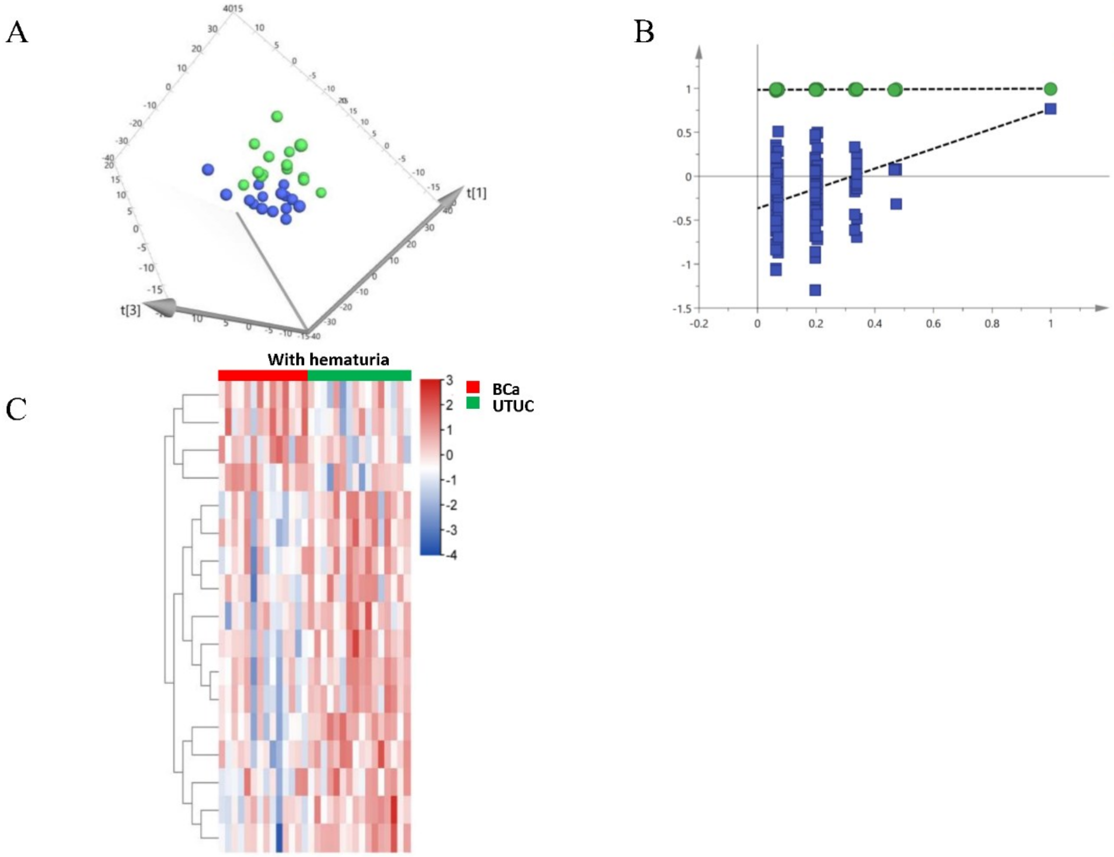

Supplementary Figure 5. | ROC curves for each metabolite selected for constructing the model. **(A).**The ROC curves of metabolites in the panel when comparing UCs and HCs. **(B).** The ROC curves of metabolites in the panel when comparing UTUC and BCa without hematuria. **(C).** The ROC curves of metabolites in the panel when comparing UTUC and BCa with hematuria.

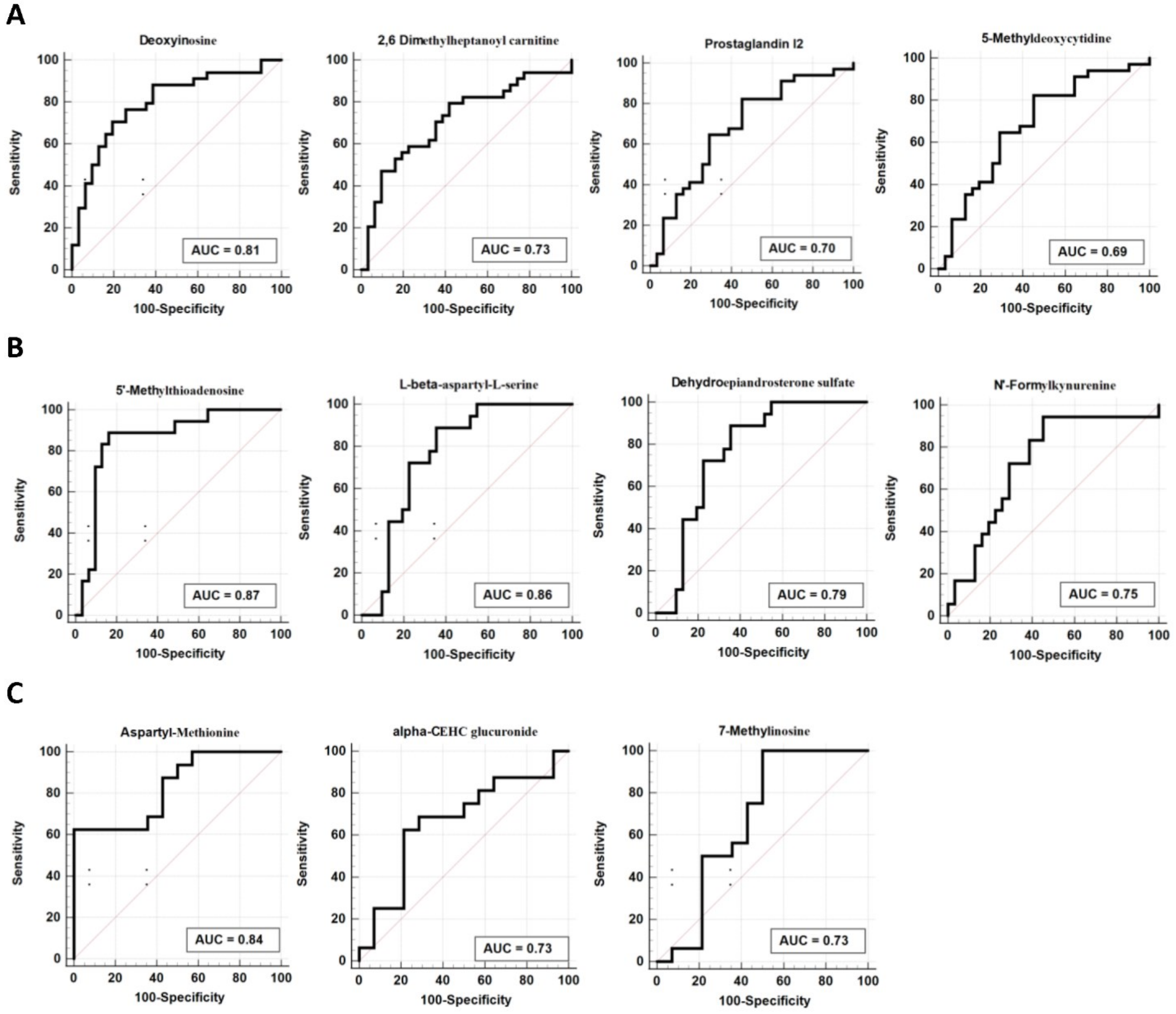

Supplementary Figure 6. | The differential metabolites both in BCa vs controls and in UC vs controls. **(A).** The intensity of prostaglandin I2 **(B).** The intensity of mystric acid **(C).** The intensity of deoxyinosine **(D).** The intensity of Tetrahydrocortisone **(E).** The intensity of 6-hydroxy-5-methoxyindole glucuronide **(F).** The intensity of 11-beta-hydroxyandrosterone-3-glucuronide

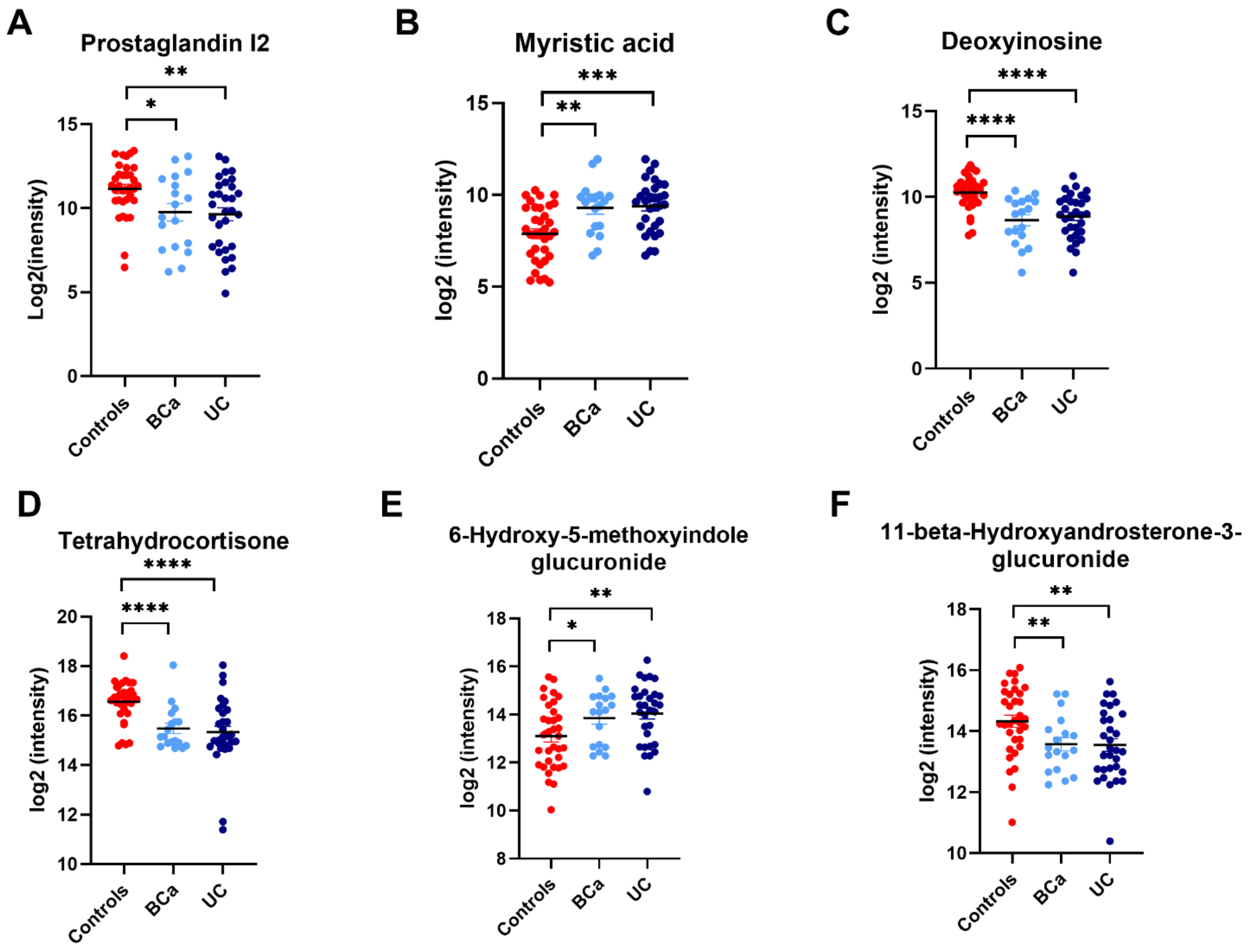

Supplement: Supplementary file 1 [file DataSheet_1.pdf]
